# Exogenous GA_3_ Promotes Germination by Reducing Endogenous Inhibitors in Sainfoin (*Onobrychis viciifolia*) Seeds

**DOI:** 10.3390/plants14101464

**Published:** 2025-05-14

**Authors:** Yanyan Luo, Kun Wang, Yuheng Yao, Lili Nan

**Affiliations:** Pratacultural College, Gansu Agricultural University, Yinmen Village, Anning District, Lanzhou 730070, China; 1073323010016@st.gsau.edu.cn (Y.L.); wangkun@st.gsau.edu.cn (K.W.); 1073324010034@st.gsau.edu.cn (Y.Y.)

**Keywords:** endogenous inhibitors, antioxidant enzyme, gibberellins, germination, seeds

## Abstract

Endogenous inhibitors can inhibit seed germination, and GA_3_ can promote seed germination. Whether GA_3_ can affect the changes in endogenous inhibitors has not been clarified. In order to study the effect of GA_3_ on the endogenous inhibitors in sainfoin (*Onobrychis viciifolia*) seeds, the systematic separation method and gas chromatography–mass pectrometry (GC-MS) method were used to determine the endogenous inhibitors using cabbage (*Brassica rapa* var. glabra Regel) as a bioassay model to validate the inhibitory activity in sainfoin seeds, and then the optimal concentration of GA_3_ was determined to promote seed germination. The results showed that endogenous inhibitors existed in the pod coat, seed coat, and seed embryo of sainfoin seeds, with the methanol and ethyl acetate phases showing the highest degree of inhibition. The organic compounds were mainly organic acids, phenols, lipids, and alkanes. The levels of changes in germination indicators, storage substances, and antioxidant enzymes determined that 600 mg/L GA_3_ was the optimum concentration to promote germination of sainfoin seed. It was also found that 600 mg/L GA_3_ reduced the relative content of endogenous inhibitors and changed the content of endogenous hormones. In summary, the presence of endogenous inhibitors may be one of the reasons for the low germination rate of sainfoin seeds, with 3-methoxycatechol and 4-nitrosodiphenylamine playing a major role. GA_3_ can reduce the relative content and types of endogenous inhibitors to promote the germination of sainfoin seeds. Our experimental results provide the basis for subsequent exploration of the mechanism of specific endogenous inhibitors and the identification of deeper molecular mechanisms.

## 1. Introduction

Sainfoin (*Onobrychis viciifolia*) is a perennial legume of the genus, which is one of the main genus of the Fabaceae family [1]. This plant has been cultivated for centuries and is predominantly found across temperate and subtropical zones, including Russia, Europe, North America, the Middle East, and northern/northeastern Africa [2]. Sainfoin is a fine animal feed resource with high nutritional value and yield, good palatability, and strong nitrogen fixation capacity [3]. Due to its high condensed tannin content in seeds, roots, stems, leaves, and flowers [4], sainfoin helps prevent bloating in ruminants while reducing urinary nitrogen excretion and enhancing amino acid absorption when used as livestock feed [5,6]. Its seeds are typically harvested within their tough, pod-like seed coats, which are difficult to remove. As a result, seeds are often sown still encased in these impermeable pods, leading to restricted water absorption and, consequently, low germination rates [7]. Since germination success directly influences sainfoin yield and quality, optimizing this stage is crucial for maximizing the crop’s agricultural potential. Thus, enhancing seed germination represents the first critical step in establishing efficient sainfoin cultivation and fully utilizing this valuable plant resource. It has been found that endogenous inhibitors are also an important factor affecting seed dormancy and germination in plants [8].

Endogenous inhibitors are organic compounds present in different parts of the plant seed, such as the pericarp, seed coat, endosperm, embryo, etc. Even if these substances are present in very small amounts in seeds, they can affect the physiological activities of seeds, such as preventing cell division, differentiation, elongation, and development, ultimately preventing seed germination [9]. There are three main sources of inhibitory substances, including those that are produced by the plant itself, such as organic acids, alkaloids, etc.; those produced by enzymatic degradation within the plant seeds in the process of plant metabolism, such as the ammonia in amygdalin is produced by the decomposition of hydrogen cyanide or nitride under the hydrolysis of amygdalin; and others produced by other plants or their seeds, such as *Artemisia absinthium* eaves secreted oil and abscistin on the surrounding plant growth, and seed germination produces allelopathic inhibition [10]. Based on their chemical structures, endogenous inhibitors can be categorized into several groups, including organic acids, volatile aromatic essential oils, esters, olefins, phenols, quinones, ketones, ethers, and gaseous compounds [11]. Cabbage (*Brassica rapa* var. glabra Regel) was selected as the standard bioassay species due to its high germination uniformity and sensitivity to inhibitors [12]. At present, there are many reports on the study of germination inhibitors in seeds, but most of the studies only stay at the level of preliminary extraction and isolation of inhibitors, and fewer of them reach the level of identification, and the study of endogenous inhibitors in seeds of leguminous plants is at the primary stage. According to the characteristics of inhibitors and their mechanism of action, different methods can be used to remove the inhibitory effect of inhibitors on plant seeds in daily agricultural and forestry production. The specific methods include physical and chemical treatments, layering, flushing, and low-temperature treatments to promote the germination of plant seeds.

GA_3_, a crucial diterpenoid plant growth regulator, plays a pivotal role in modulating various developmental processes including seed germination, root development, stem elongation, and fruit maturation [13]. This phytohormone exerts its effects by stimulating cell division and elongation, thereby accelerating leaf bud growth. Additionally, GA_3_ breaks seed dormancy and enhances germination rates through multiple mechanisms: it degrades the seed cuticle wax layer to improve water permeability and gas exchange while simultaneously increasing respiratory activity to facilitate metabolic processes essential for germination [14]. Little is known about the effects of endogenous inhibitors in plant seeds during GA_3_-induced seed germination.

Therefore, sainfoin seeds were used as experimental materials in this study. The extraction liquid of various parts of GA_3_-treated sainfoin seeds was separated by systematic solvent method, and the composition of endogenous inhibitors was determined by gas chromatography–mass spectrometry (GC-MS), so as to explore the relationship between exogenous GA_3_ and endogenous inhibitors. The study was to provide reference for further research on dormancy and germination mechanism of sainfoin seeds.

## 2. Materials and Methods

### 2.1. Plant Materials

In this study, sainfoin (*O. viciifolia* cv. Gansu) and cabbage *(Brassica rapa* var. glabra Regel) seeds were used as experimental materials. *O. viciifolia* cv. Gansu seeds were provided by Pratacultural College of Gansu Agricultural University. *Brassica rapa* var. *glabra* Regel seeds were purchased from Hezhiyuan Seed Company, with 96% purity, 85% germination rate, and 7% moisture. Cabbage seeds germinate quickly and neatly and are used only as bioassay models for validating the activity of endogenous inhibitors.

### 2.2. Extraction of Endogenous Inhibitors from Different Parts of Sainfoin Seeds

The sainfoin seeds were soaked in distilled water (as a control) and 600 mg/L GA_3_ solution for 24 h. The pod skin, seed coat, and seed embryo were then separated. The pod skin, seed coat, and seed embryo were weighed at 5 g each, grounded to a powder in a pre-cooled mortar, and added to 50 mL 80% methanol solution at 4 °C for 24 h. The extracts were repeated three times with stirring at intervals, and the extracts were combined. Following centrifugation at 4000× *g* r/min for 10 min the supernatant was reserved. The supernatant was concentrated under reduced pressure at 65 °C, and finally the concentrate was fixed to 10 mL with distilled water to obtain the methanol extract of each part.

### 2.3. Preliminary Isolation of Endogenous Inhibitors from Sainfoin Seeds

**Figure 1:**
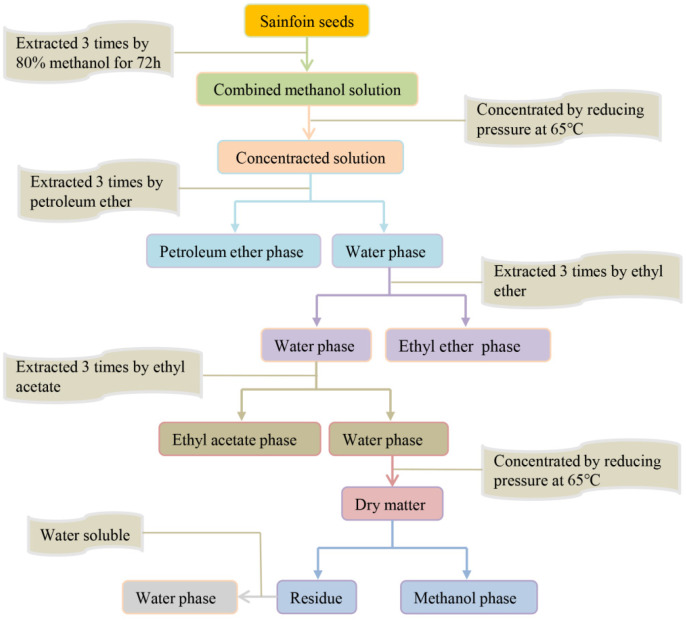
A systematic solvent separation process for endogenous inhibitory substances in sainfoin seeds.

The methanol extracts were separated into 5 fractions (petroleum ether phase, ethyl ether phase, ethyl acetate phase, methanol phase and water phase). As shown in Figure 1, firstly, 10 mL of petroleum ether was added to the methanol extract, repeated 3 times, and the extracts were combined, which was the petroleum ether phase of each part of the inhibitor; 10 mL of ethyl ether was added to water phase, repeated 3 times, and the extract was combined, which was the ethyl ether phase of each part of the inhibitor; 10 mL of ethyl acetate was added to water phase, repeated 3 times, and the extract was combined, which was the ethyl acetate phase of each part of the inhibitor. Finally, the water phase of each part of the inhibitor was reduced in pressure, concentrated, and dried at 65 °C; the dry matter was dissolved with methanol and filtered, and the filter residue was dissolved with water to obtain the methanol phase and water phase of each part of the inhibitor. Then, the organic phase solutions were evaporated and concentrated on a rotary evaporator, and finally the organic phase was concentrated to 10 mL and stored in a refrigerator at 4 °C for spare parts.

### 2.4. Determination of the Activity of Each Isolated Phase Using Cabbage

The full and uniformly sized seeds of cabbage (*Brassica rapa* var. glabra Regel) were sterilized with 0.1% HgCl_2_ solution for 5 min and rinsed several times with sterile water. The 3 mL of organic solvent was added to a Petri dish with a diameter of 9 cm and two layers of filter paper after the organic solvent evaporated. The cabbage seeds were placed in a Petri dish and added with 5 mL of distilled water. Only distilled water was added to the control (CK). Petri dishes were placed in a light incubator at a temperature of 25 °C and a relative humidity of 80% for germination tests. To keep the petri dish moist, the germination bed is sprayed with distilled water daily. The germination rate of cabbage seeds was measured after 48 h, and the root length and seedling height of cabbage were measured after 72 h. All germination experiments had six biological replicates of 50 seeds each.

### 2.5. Determination of the Chemical Composition of Each Isolated Phase in the Sainfoin Seeds

A concentrated sample of 2 mL of methanol and ethyl acetate phases were sent to the State Key Laboratory of Aridland Crop Science of Gansu Agricultural University for the identification of the components by GC-MS. The instrument model is Agilent 7000D-7890B GC-MS. The GC conditions were a HP-1 quartz capillary column of 30 m × 250 μm × 0.25 μm, injection temperature of 280 °C, the carrier gas was high purity helium, and the temperature of the gasification chamber was 325 °C. The MS conditions were electron ionization (EI) source, an ion source temperature of 230 °C, ionization voltage of 70 eV, a collection current of 300 μA, an emission current of 150 mA, and a scanning range of 29–500. Finally, the mass spectra of each component was checked by a computer-controlled inventory signal and verified with the standard spectrogram, and the total ion flow chromatogram of each organic phase was obtained. The total ions were integrated, and the peak area normalization method was used to obtain the proportion of total mass accounted for by each organic substance.

### 2.6. Sainfoin Seed Germination and Measurement of Physiological Indicators

The full and uniformly sized seeds of *O. viciifolia* cv. Gansu were sterilized with 10% NaClO solution for 10 min and rinsed several times with sterile water. Then, the seeds were soaked in 0, 200, 400, 600, and 800 mg/L GA_3_ solution for 24 h, respectively. The soaked seeds were placed in a Petri dish with a diameter of 9 cm and two layers of filter paper and added with distilled water. Petri dishes were placed in a light incubator at a temperature of 25 °C and a relative humidity of 80% for germination tests. To keep the Petri dish moist, the germination bed was sprayed with distilled water daily. The sainfoin seeds with radicles greater than 2 mm were counted to calculate germination rate. The plumule and radicle length of germinated seeds were determined, soluble protein content was determined using the coomassie brilliant blue G-250 staining method, soluble sugar and starch content were determined using the anthrone colourimetric method, superoxide dismutase (SOD) activity was determined using the nitrotetrazolium blue chloride (NBT) method, catalase (CAT) activity was determined using the potassium permanganate titration method, and peroxidase (POD) activity was determined by the guaiacol method [15]. All germination experiments had six biological replicates of 30 seeds each. The content of ZT, IAA, GA_3_ and ABA in pod skin, seed coat and seed embryo was identified using high performance liquid chromatography (HPLC) [16].

### 2.7. Statistical Analysis

SPSS 22.0 software was used to perform one-way analysis of variance (ANOVA) on the experimental data. Excel 2010 was used to analyze and calculate the data. Duncan’s test was used to analyze the difference significance (*p* < 0.05).

## 3. Results

### 3.1. Determination of the Biological Activities of the Isolated Phases Using Cabbage

**Figure 2:**
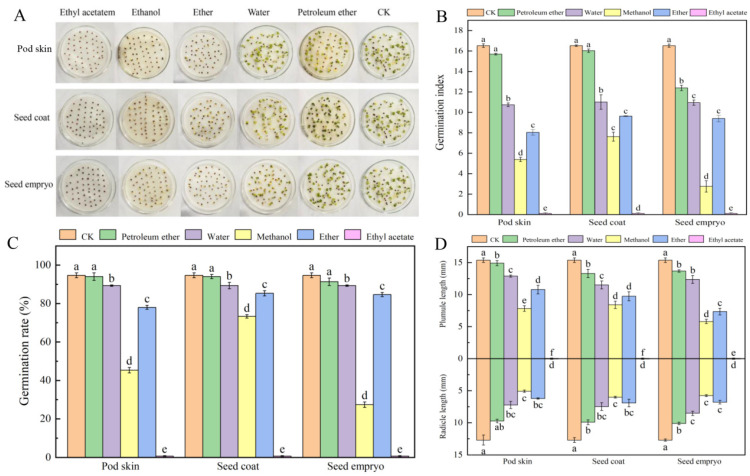
Effect of each isolate phases of pod skin, seed coat and seed embryo of sainfoin on cabbage germination. (**A**) Effect of each isolate phases on seed germination phenotype of cabbage. (**B**) Effect of each isolate on seed germination index. (**C**) Effect of each isolate phases on seed germination rate. (**D**) Effect of each isolate phases on plumule and radicle length. Error bars represent standard deviation (SD) of six replicates. Bars with different lowercase letters were significantly different by Duncan’s multiple range test (*p* < 0.05). CK represents the control.

Cabbage seeds germinate quickly and neatly, so they are often used to verify the biological activity of the extracted substances in each isolation phase. In this study, the effects of each isolation phase on the germination of cabbage seeds was shown in Figure 2. The preliminary test showed that the petroleum ether, ether, ethyl acetate, and methanol had no significant effect on the germination of cabbage seeds on their own. Therefore, the changes in the germination rate, germination index, and plumule and radicle length of cabbage seeds after treatment with different isolated phases were all caused by endogenous inhibitors in the extract. Compared with CK, the ether phase, ethyl acetate phase, methanol phase, and water phase of the sainfoin pod skin all had significant inhibitory effects on the germination of cabbage seeds (Figure 2A,C), and the inhibitory effect of ethyl acetate phase was the strongest, and the cabbage seeds did not germinate. The second was methanol phase, with the germination rate decreasing to 45.33%, which was 52.12% lower than that of CK (94.67%); the ether phase was again followed by the methanol phase, with the germination rate decreasing to 78%, which was 17.61% lower than that of CK, and then the germination rate of water phase decreased to 89.33%, which was 5.64% lower than that of CK. The germination rate of cabbage in the petroleum ether phase had a small decrease, but the decrease was not significant compared with CK. In the seed coat of sainfoin seed, compared with CK (94.67%), the germination rate of cabbage seeds was decreased by 22.54%, 9.87%, and 5.64% with methanol phase, ether phase, and water phase extracted, respectively. The germination rate of cabbage seeds was completely inhibited by ethyl acetate phase, but there was no significant change in petroleum ether phase. Compared with CK (94.67%), the germination rate of cabbage seeds was decreased by 71.13%, 10.56%, and 70.34% with methanol phase, ether phase, and water phase extracted from the seed embryo of sainfoin seed, respectively. The above results showed that the extracts of pod skin, seed coat, and seed embryo had obvious inhibitory effect on the germination of cabbage seeds, and the inhibitory degree of the extracts on the germination rate of cabbage seeds was as follows: ethyl acetate phase > methanol phase > ether phase > water phase > petroleum ether phase.

The germination index and plumule and radicle lengths of cabbage seeds followed the same trend with germination rate (Figure 2B,D). Among them, the germination index, plumule and radicle length of methanol phase in pod skin decreased by 67.37%, 49.02%, and 60.03%, respectively, compared with CK. The results of germination rate, germination index, and plumule and radicle length of cabbage seeds indicated that there might be some germination inhibiting substances in the ethyl acetate, methanol, and ether isolate phases of sainfoin seeds.

### 3.2. Identification of the Chemical Composition of the Isolated Phases from Sainfoin Seeds

**Figure 3:**
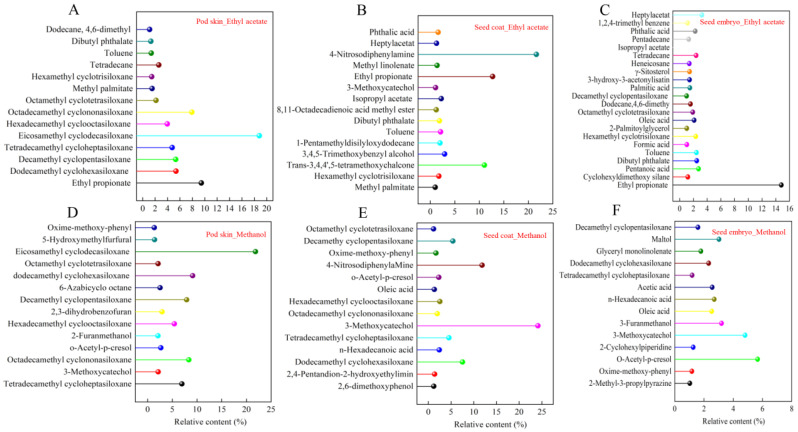
Compositional analysis of endogenous inhibitors in ethyl acetate and methanol phases of sainfoin seeds. (**A**) Relative content of endogenous inhibitors of ethyl acetate phase in pod skin. (**B**) Relative content of endogenous inhibitors of ethyl acetate phase in seed coat. (**C**) Relative content of endogenous inhibitors of ethyl acetate phase in seed embryo. (**D**) Relative content of endogenous inhibitors of methanol phase in pod skin. (**E**) Relative content of endogenous inhibitors of methanol phase in seed coat. (**F**) Relative content of endogenous inhibitors of methanol phase in seed embryo. Different colors represent different organic compounds.

According to the different degrees of inhibition of cabbage seed germination, the total ion flow chromatogram of the extract solution of pod skin, seed coat, and seed embryo were obtained by GC-MS analysis of the ethyl acetate phase and methanol phase with high inhibitory activity. The peak of the total ion flow chromatogram with large peak area and high similarity was retrieved by the mass spectrometry system and verified with the standard chromatogram. These organic compounds were listed in Table S1. As can be seen from Figure 3, 14 organic compounds (relative content greater than 1) were identified in the extracts of the ethyl acetate phase and methanol phase of the pod skin, respectively (Figure 3A,D), of which the absolute dominant ones were eicosamethyl cyclodecasiloxane, ethyl propionate, octadecamethyl cyclononasiloxane, dodecamethyl cyclohexasiloxane, decamethyl cyclopentasiloxane, tetradecamethyl cycloheptasiloxane, and hexadecamethyl cyclooctasiloxane. In total,15 and 14 organic compounds (relative content greater than 1) were identified in the ethyl acetate phase and methanol phase extracts of the seed coat, respectively (Figure 3B,E), of which the absolute dominant ones were 4-Nitrosodiphenylamine, ethyl propionate, trans-3,4,4′,5-tetramethoxychalcone, 3-methoxycatechol, dodecamethyl cyclohexasiloxane, and decamethy cyclopentasiloxane. In total, 22 and 21 organic compounds (relative content greater than 1) were identified in the ethyl acetate phase and methanol phase extracts of the seed embryos, respectively (Figure 3C,F), of which the absolute dominant ones were ethyl propionate, 2,3-dihydrobenzofuran, methyl acetate, o-Acetyl-p-cresol, and 6-Azabicyclo octane. The above results showed that the compounds detected in the seed pods were mainly organosilicon compounds, lipids, alkanes, aldehydes, and alcohols. The compounds detected in the seed embryo were mainly organic acids, lipids, phenols, and aromatics. The compounds detected in the seed coat were mainly ketones, phenols, anilines, and organosilicon compounds.

### 3.3. Effect of GA_3_ on the Seed Germination of Sainfoin

**Figure 4:**
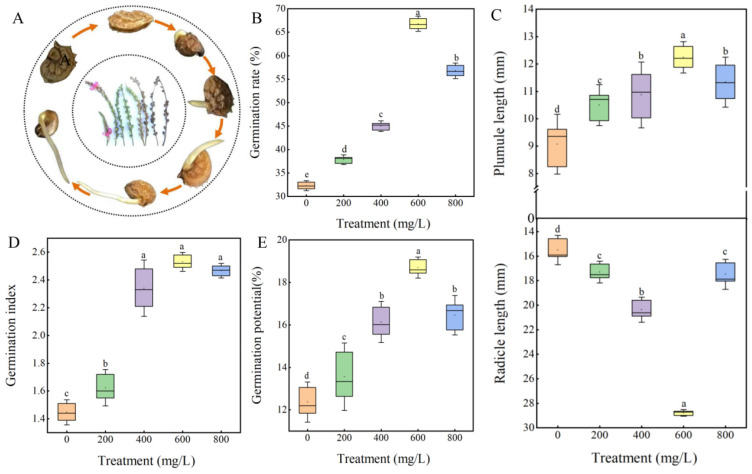
Effect of different concentrations of GA_3_ on germination of sainfoin seeds. (**A**) Dynamic process of seed germination in sainfoin. (**B**) Effect of GA_3_ treatment on seed germination rate. (**C**) Effect of GA_3_ treatment on plumule and radicle length. (**D**) Effect of GA_3_ treatment on seed germination index. (**E**) Effect of GA_3_ treatment on seed germination potential. Error bars represent standard deviation (SD) of six replicates. Bars with different lowercase letters were significantly different by Duncan’s multiple range test (*p* < 0.05).

In order to explore the method of eliminating germination inhibitors in sainfoin seeds, the study used different concentrations of GA_3_ to treat seeds (Figure 4). The results showed that 200 mg/L, 400 mg/L, 600 mg/L, and 800 mg/L GA_3_ treatments significantly increased the germination rate of sainfoin seeds compared with the control. Among them, the 600 mg/L GA_3_ was the most effective, with 66.67% seed germination rate, which was 42.51% higher compared to the control (38.33%) (Figure 4B). The germination potential, germination index, and plumule and radicle lengths also reached their maximum at 600 mg/L (Figure 4C–E), which increased by 34.37%, 44.18%, 11.06%, and 44.63%, respectively, compared to the control. These results indicated that exogenous GA_3_ significantly increased seed germination as well as the growth of plumule and radicle.

### 3.4. Effect of GA_3_ on Physiological Indexes of Sainfoin Seeds

**Figure 5:**
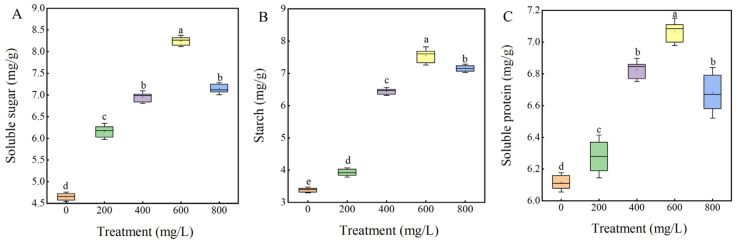
Effects of different concentrations of GA_3_ treatments on contents of (**A**) soluble sugars, (**B**) starch, and (**C**) soluble protein in germinating seeds of sainfoin. Error bars represent standard deviation (SD) of three replicates. Bars with different lowercase letters were significantly different by Duncan’s multiple range test (*p* < 0.05), same below.

**Figure 6:**
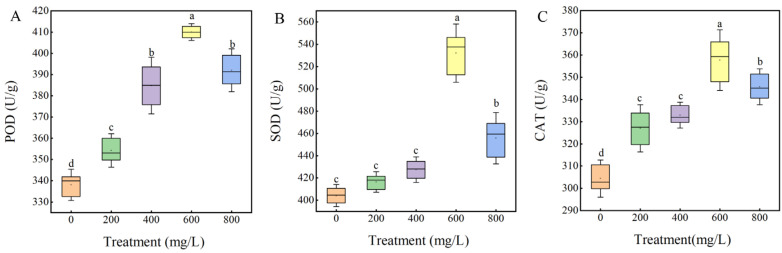
Effects of different concentrations of GA_3_ treatments on activities of (**A**) POD, (**B**) SOD, and (**C**) CAT in germinating seeds of sainfoin.

GA_3_ treatment significantly improved the germination parameters of sainfoin seeds. In order to assess the effect of exogenous GA_3_ on seed physiological indexes, the present study was conducted to investigate the changes in the content of storage substances and antioxidant enzyme activities in GA_3_-treated sainfoin seeds (Figure 5 and Figure 6). GA_3_-treated seeds accumulated more soluble sugars, soluble proteins and starch than the control (Figure 5). With the increase in treatment concentration, the contents of soluble sugars, soluble proteins, and starch firstly increased and then decreased and then reached the maximum value at 600 mg/L, which were increased by 42.92%, 14.12%, and 54.73%, compared to control, respectively (*p* < 0.05). Other concentrations also showed significant changes compared to the control. The above results indicated that 600 mg/L GA_3_ significantly increased the accumulation of storage material and provided more energy for the germination of sainfoin seeds.

As can be seen from Figure 6, different concentrations of GA_3_ treatment of sainfoin seeds significantly increased the antioxidant enzyme activities. With the increase in treatment concentration, the activities of POD, CAT, and SOD firstly increased and then decreased. The best effect was obtained at 600 mg/L GA_3_ treatment, which POD, CAT, and SOD increased by 16.82%, 15.88%, and 25.19%, respectively, and the difference was significant (*p* < 0.05). In contrast, the POD, CAT, and SOD activities in control sainfoin seeds changed slowly. The analysis of the results indicated that the appropriate concentration of GA_3_ increased the activity of antioxidant enzymes within the seeds, which could effectively remove the reactive oxygen radicals generated during the seed germination process, thus promoting seed germination. Therefore, 600 mg/L GA_3_ was selected for subsequent experiments.

### 3.5. Effect of GA_3_ on Endogenous Inhibitors in Sainfoin Seeds

**Figure 7:**
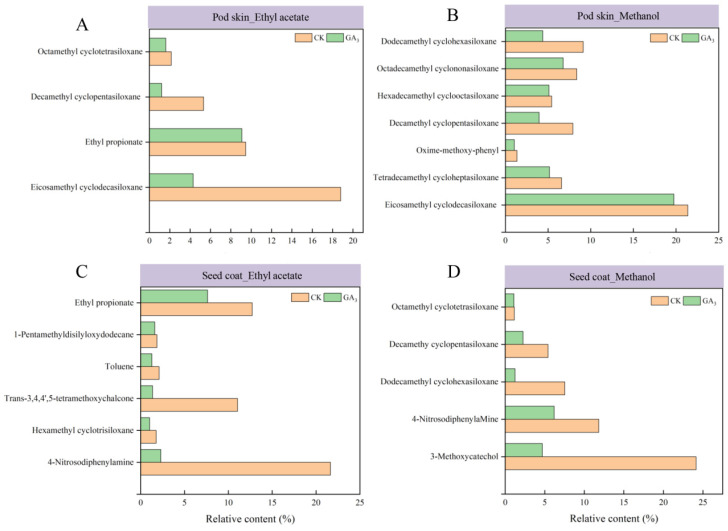
Effect of GA_3_ treatment on endogenous inhibitors in seeds of sainfoin. (**A**) Relative content of endogenous inhibitors of ethyl acetate phase in pod skin. (**B**) Relative content of endogenous inhibitors of methanol phase in pod skin. (**C**) Relative content of endogenous inhibitors of ethyl acetate phase in seed coat. (**D**) Relative content of endogenous inhibitors of methanol phase in seed coat.

The above results indicated that 600 mg/L GA_3_ had the most positive effect on the germination of sainfoin seeds, but could 600 mg/L GA_3_ attenuate or eliminate the inhibitory effect of endogenous inhibitors in sainfoin seeds? Based on the results of the previous experiments, the extracts of methanol and ethyl acetate phases with stronger inhibition were identified by GC-MS. As can be seen from the experimental results, the species of endogenous inhibitors in pod skin, seed coat and seed embryo were reduced compared to CK after GA_3_ treatment (Tables S2–S4). There were four identical endogenous inhibitors in the pod skin_ethyl acetate phase and seven identical endogenous inhibitors in the pod skin_methanol phase of the CK and GA_3_ treatments, and the relative content of endogenous inhibitors was reduced in the GA_3_ treatment compared to CK (Figure 7A,B). There were six identical endogenous inhibitors in the seed coat_ethyl acetate phase and five identical endogenous inhibitors in the seed coat_methanol phase, in which 3-Methoxycatechol and 4-Nitrosodiphenylamine decreased the most, by 80.56% and 98.93%, respectively, compared to CK (Figure 7C,D). After GA_3_ treatment, the types of endogenous inhibitors were significantly reduced in seed embryo. The above results indicate that GA_3_ reduces the relative content and type of endogenous inhibitors, thus attenuating the degree of inhibition to promote the germination of sainfoin seeds. Based on the reduction in relative content, 3-methoxycatechol and 4-nitrosodiphenylamine were ultimately identified as the primary endogenous inhibitors. Further research will be conducted to understand their inhibitory effects and functions.

### 3.6. Effect of GA_3_ on Endogenous Hormones in Sainfoin Seeds

**Figure 8:**
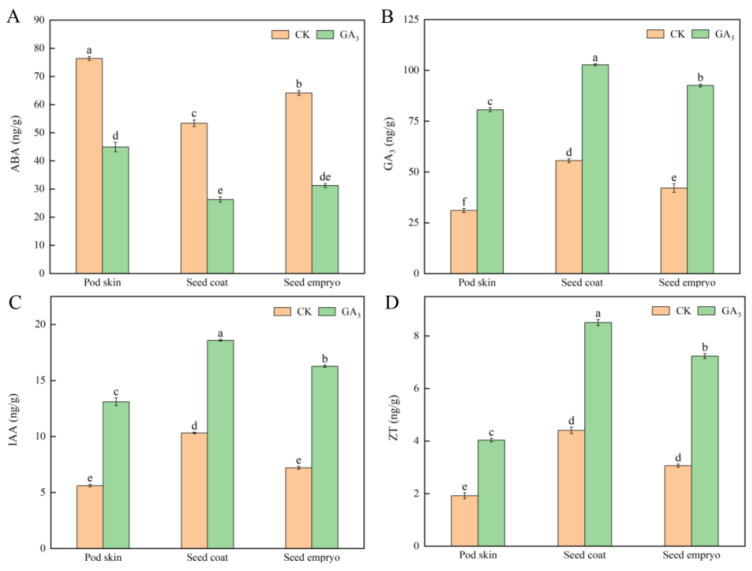
Changes in the contents of (**A**) ABA, (**B**) GA_3_, (**C**) IAA, and (**D**) ZA endogenous hormones in pod skin, seed coat, and seed embryo during GA_3_ treatment. The content of ZT, IAA, GA_3_, and ABA was identified using HPLC from sainfoin seeds.

In order to more clearly explain the effect of phytohormones on the germination of sainfoin seeds, we analyzed the contents of ABA, GA_3_, IAA, and ZA in the pod skin, seed coat, and seed embryo (Figure 8). The results showed that after GA_3_ treatment, the contents of ZA, GA_3_, and IAA were significantly increased compared to the control, while the contents of ABA were significantly decreased. The content of ABA decreased by 42.23%, 45.67%, and 51.57% in the pod skin, seed coat, and seed embryo, respectively. It was found that ABA content in pod skin and seed embryo was higher than that in seed skin, while ZA, GA_3_, and IAA contents showed an opposite trend, indicating that 600 mg/L GA_3_ promoted germination by reducing ABA content and increasing ZA, GA_3_, and IAA contents in pod skin, seed coat, and seed embryo to reduce the degree of seed inhibition. The change trend was consistent with that of endogenous inhibitors.

## 4. Discussion

### 4.1. Sainfoin Seeds Contain Endogenous Inhibitors

An important factor for the low and unstable seed germination rate is the presence of inhibitory compounds in the outer seed coat or middle hard layer of the seed coat [17], such as abscisic acid (ABA), salicylic acid (SA), and phenolic and lipid compounds. It can induce seed dormancy and prevent germination [18]. The inhibitory compounds in the embryo will produce physiological inhibition, hindering the growth potential or breakthrough ability of the embryo, which directly lead to the seed embryo being unable to break the mechanical resistance of the solid structure such as the pod skin, thus affecting the seed germination [19]. It was found that the seed coat of *Sapium sebiferum* contained a high concentration of germination inhibitors, and the seeds were affected by the inhibitory substances. It takes a long time to complete germination [20]. In this study, according to the polarity of the organic solvent, the endogenous inhibitor of the pod skin, seed coat, and seed embryo was extracted, respectively, to study the biological activity on cabbage as a receptor plant. Because of the rapid and orderly germination of cabbage seeds, it is often used to analyze the biological activity of endogenous inhibitors. The results showed that the pod skin, seed coat and seed embryo of sainfoin contained organic compound to inhibit the germination of cabbage seeds, among which the ethyl acetate phase and methanol phase extract showed the most obvious inhibitory effect. The extract not only inhibited the germination of cabbage seeds, but also significantly shortened the length of plumule and radicle. Similar results were reported for inhibitors isolated from seeds of *Sapium sebiferum* [21].

In recent years, with the deepening of research on seed germination disorders, the identification of endogenous inhibitors has become a part of the mechanism of seed germination. The ethyl acetate phase and methanol phase extracts with the strongest inhibitory effect were detected by GC-MS, which were mainly organic acids, esters, alkanes, and ketones. These compounds may have stronger inhibitory effects, thus inhibiting the germination of cabbage seeds. There is considerable variation in the composition of germination inhibitors among different plant seeds. It was found that proanthocyanidins (PAs) contained in the seed skin and embryo of *Sapium sebiferum* were the main inhibitory substances affecting seed germination [20]. The amides and alcohols identified in the seed extract of *Crataegus viridis* were the main substances causing seed dormancy [22]. In addition, specific substances such as dimethylcyclopentane, eugenol, coumarin, and vanillic acid were all considered to inhibit seed germination and growth [23]. This result is different from the compounds identified in other plant seeds by others [24], which may be due to the fact that different plant seeds produce different inhibitory substances to adapt to different environments. Some plant seeds are used to ward off insect food, some to prevent waterlogging or drought, and most plant seeds to avoid germination in cold environments. These plants produce many different inhibitors in their seeds for different reasons but may play a major role only some of them [25]. The isolation and identification of the endogenous inhibitors of sainfoin seeds have not been reported, and the mechanism and mode of action of these endogenous inhibitors are still unclear.

### 4.2. GA_3_ Promotes Sainfoin Seed Germination by Altering the Content Levels of Storage Substances and Antioxidant Enzymes

Plant hormones can affect seed germination [26,27,28]. Exogenous GA_3_ treatment can significantly promote the germination of plant seeds [29,30]. As the optimal concentration varies with different species, 200 mg/ L, 400 mg/ L, 600 mg/ L, and 800 mg/ L GA_3_ were used to treat sainfoin seeds in this study. Compared with CK, GA_3_ could improve the germination rate and plumule and radicle length of sainfoin seeds. Among them, 600 mg/L GA_3_ had the most significant promoting effect. It was also found that 200 mg/L GA_3_ effectively improved the germination rate of *Acer mono* seeds [31]. The concentration of 150 mg/L GA_3_ resulted in the highest germination rate, germination index, and vitality index of *Nitraria tangutorum* seeds [32]. The optimal concentration of GA_3_ varies due to species differences. Therefore, our experimental results suggest that 600 mg/L GA_3_ may be a good choice to promote the germination of sainfoin seeds.

The storage substances, namely soluble sugar, soluble protein and starch, provide energy and nutrition for seed germination, and the change in storage substance content represents the change in seed germination process and metabolic activity. In our study, GA_3_ (200–800 mg/L) treatment increased the level of stored substances, with 600 mg/L GA_3_ having the most significant boosting effect. Therefore, our results provide further evidence that 600 mg/L GA_3_ is the optimal concentration to promote sainfoin seed germination. Seed dormancy and germination are not only related to the decomposition and utilization of stored substances but also have different changes in antioxidant enzymes during this process [33]. The internal respiratory metabolism of seeds will be enhanced with the release of dormancy to germination, resulting in excessive accumulation of reactive oxygen species and osmotic substances, which will further cause oxidative damage to cell structure [34,35] and ultimately lead to the increase in the content of malondialdehyde, the product of lipid peroxidation in the cell membrane, which has long been studied and confirmed in the process of seed germination [36]. SOD can react quickly to reduce toxicity [37], CAT and POD can cooperate to remove a large number of reactive oxygen species, and antioxidant enzymes can effectively reduce and remove the damage of free radicals to cell membranes [38]. Zhang Lili et al. showed that exogenous GA_3_ treatment of rice seeds could increase SOD and POD activities during the seed process [39]. The results of this experiment showed that different concentrations of GA_3_ could significantly improve the activities of SOD, POD, and CAT during seed germination, effectively remove the damage of reactive oxygen species to cells, and promote seed germination.

### 4.3. GA_3_ May Regulate the Levels of Endogenous Inhibitors and Endogenous Hormones of Sainfoin Seeds

Most germination inhibitors are natural compounds produced by dormant seeds under natural conditions, such as ABA and 1,2,3-benzenetriol produced in *Cercis chinensis* seeds to regulate their dormancy [12]. Through gibberellin pretreatment and stratification experiments, germination capacity is improved by reducing or attenuating germination inhibitors in the seed [40,41]. In this study, 600 mg/L GA_3_ reduced the relative content and type of endogenous inhibitors. The results of this study were consistent with the study of Chen et al., who found that coumarin delayed germination of *Brassica parachinensis* seeds and reduced the content of GA_4_ in seeds. Exogenous GA_4_ also restored the germination delay caused by coumarin [42]. In earlier studies, Eiuhellin pointed out that phenols have an inhibitory effect on the metabolism of key enzymes for seed germination and can also affect the growth of seedlings [43]. Coumarin can also affect the activity of antioxidant enzymes in rice and inhibit ABA catabolism to inhibit rice germination [44]. The observed substantial decrease in 3-methoxycatechol and 4-nitrosodiphenylamine levels may represent a key mechanism through which GA_3_ enhances sainfoin seed germination. 4-Nitrosodiphenylamine, an aniline derivative, exhibits strong germination-inhibiting properties, while 3-methoxycatechol (a dihydroxyl phenolic compound) demonstrates moderate inhibitory activity, consistent with the established inverse relationship between phenolic OH group abundance and inhibitory potency [45]. These findings align with previous reports that seed coat phenolics, like those in *Cornus officinalis*, can impede oxygen diffusion and suppress germination [46]. Although this study reveals GA_3_’s influence on endogenous inhibitors, the precise mechanisms underlying exogenous GA_3_-mediated reduction in these compounds remain unclear, warranting further investigation. The results nevertheless provide important insights into the phytohormonal regulation of germination inhibitors in sainfoin.

Hormones are the main members of signal regulation in plants. Almost all plant hormones, such as ABA, ZA, IAA, GA, JA, CTK, SA, and ETH, their molecular synthesis, metabolic pathways, and intermolecular interactions played an important role in seed germination [47]. Studies have shown that IAA is an important signaling molecule that has synergistic or opposite effects with other hormones during plant growth, and collaborates with ABA to promote seed dormancy [48,49]. GA_3_ can break the mechanical restraint of seed coat and promote the radicle protrusion during seed germination [50]. ABA regulates the accumulation of chemicals in seeds and controls dehydration in later seed development [51]. The content of ABA in dry seeds was relatively high and decreased rapidly after germination [52,53]. In addition, since ABA prevents endosperm rupture, the key of seed completely germination is to minimize the amount of endogenous ABA [54]. Exogenous GA_3_ promoted seed germination by reducing the inhibitory effect of ABA [55]. ZA mainly exists in seeds, has strong metabolism and antagonistic effect on seed germination inhibitors [56]. In this study, 600 mg/L GA_3_ treatment decreased the content of ABA, increased the contents of GA_3_, ZA, and IAA, and decreased the relative content and types of endogenous inhibitors, so as to promote seed germination.

In this study, only the types and relative contents of endogenous inhibitory substances in the pod skin, seed coat, and seed embryo of GA_3_-treated sainfoin seeds were measured. The endogenous inhibitors produced loss or transformation in different ways in the process of GA_3_-induced seed germination, so that the seeds could finally break the shell and germinate successfully. The specific transformation mode needs to be further confirmed by experiments. In view of these results, combined with previous studies on the relationship between germination inhibitors, GA, antioxidant enzyme activities, and seed germination, we conclude that GA_3_ regulates seed germination by modulating the synthesis of endogenous inhibitors, thereby affecting antioxidant enzyme activity and hormone content.

## Data Availability

The original contributions presented in this study are included in the research paper and in the Supplementary Materials. Further inquiries can be directed to the corresponding authors.

## Supplementary Materials

The following supporting information can be downloaded at: https://www.mdpi.com/article/10.3390/plants14101464/s1, Table S1: Endogenous inhibitors extracted from each isolated phase of sainfoin seeds; Table S2: Endogenous inhibitor extracted from the methanol and ethyl acetate phases of the pod skin of sainfoin and their relative contents; Table S3: Endogenous inhibitor extracted from the methanol and ethyl acetate phases of the seed embryo of sainfoin and their relative contents; Table S4: Endogenous inhibitor extracted from the methanol and ethyl acetate phases of the seed embryo of sainfoin and their relative contents.
